# Storage Property Is Positively Correlated With Antioxidant Capacity in Different Sweet Potato Cultivars

**DOI:** 10.3389/fpls.2021.696142

**Published:** 2021-11-23

**Authors:** Hui-Hui Song, Zhi-Lin Zhou, Dong-Lan Zhao, Jun Tang, Yan-Hong Li, Zhuo Han, Xiao-Yan Chen, Kang-Di Hu, Gai-Fang Yao, Hua Zhang

**Affiliations:** ^1^School of Food and Biological Engineering, Hefei University of Technology, Hefei, China; ^2^Xuzhou Institute of Agricultural Sciences of the Xuhuai District of Jiangsu Province, Xuzhou, China

**Keywords:** sweet potato, storage property, antioxidant capacity, reactive oxygen species (ROS), correlation analysis

## Abstract

Sweet potato decays easily due to its high respiration rate and reactive oxygen species (ROS) accumulation during postharvest storage. In this study, we explored the relationship between antioxidant capacity in leaves and storage properties in different sweet potato cultivars, the tuberous roots of 10 sweet potato cultivars were used as the experimental materials to analyze the storage property during storage at 11–15°C. According to the decay percentage after 290 days of storage, Xu 32 was defined as a storage-tolerant cultivar (rot percentage less than 25%); Xu 55-2, Z 15-1, Shangshu 19, Yushu, and Zhezi 3 as above-moderate storage-tolerant cultivars (rot percentage ranging from 25 to 50%); Sushu 16, Yanshu 5, and Hanzi as medium-storable cultivars (rot percentage 50–75%); and Yan 25 as a storage-sensitive cultivar (rot percentage greater than 75%). Meanwhile, analysis of the α-amylase activity in root tubers of the 10 sweet potato cultivars during storage indicated that α-amylase activity was lowest in the storage-tolerant cultivar Xu 32 and highest in the storage-sensitive cultivar Yan 25. Evaluation of antioxidant enzyme activities and ROS content in the leaves of these 10 cultivars demonstrated that cultivar Xu 32, which showed the best storage property, had higher antioxidant enzyme activity [superoxide dismutase (SOD), catalase (CAT), ascorbate peroxidase (APX), and peroxidase (POD)] but lower lipoxygenase (LOX) activity, hydrogen peroxide (H_2_O_2_) and malondialdehyde (MDA) contents, and superoxide anion radical (O_2_⋅^–^) production rates compared with those of the storage-sensitive cultivar Yan 25 and the medium-storability cultivars Hanzi, Yanshu 5, and Sushu 16. Additionally, principal component analysis (PCA) suggested that sweet potato cultivars with different storage properties were clustered separately. Correlation and heat map analysis further indicated that CAT, APX, POD, and SOD activities were negatively correlated with α-amylase activity, while LOX activity and MDA and H_2_O_2_ contents were negatively correlated with the storage property of sweet potato. Combined, our findings revealed that storage property is highly correlated with antioxidant capacity in sweet potato leaves and negatively correlated with α-amylase activity in tuberous roots, which provides a convenient means for the screening of storage-tolerant sweet potato cultivars.

## Introduction

Sweet potato (*Ipomoea batatas* L.), which was domesticated in tropical America, is gradually becoming one of the main food crops worldwide (Mwanga et al., 2017). According to the Food and Agriculture Organization (FAO) of the United Nations, global sweet potato production exceeded 140 million tons in 2019, with China accounting for the largest plantation area (FAO, 2019). Sweet potatoes are rich in many nutrients such as vitamins, dietary fiber, and minerals, as well as other ingredients that are beneficial to human health, including flavonoids, carotenoids, and anthocyanins (Wang et al., 2016; Kang et al., 2017). Additionally, starch is the major component of the storage root of sweet potato, accounting for 50–80% of its dry matter (Zhang et al., 2017). Amylase activity has been reported to change in sweet potato roots during storage (Takahata et al., 1995; Zhang et al., 2002). Sweet potato tubers are relatively difficult to store long-term due to their high moisture content and respiration rate, as well as the deterioration of the quality of its flesh during postharvest (Sugri et al., 2017). During postharvest storage, endogenous α-amylase and β-amylase enzyme activities influence the starch structure and reduce the starch content, which greatly affects the commodity value of this tuberous root (Lu et al., 2020). Sweet potato is also susceptible to chilling injury owing to its tropical origins (Li et al., 2018). Combined, these observations are indicative of the importance of postharvest storage for the industrial application of sweet potatoes.

Postharvest senescence includes the loss of texture, membrane injury, and decay (Ali et al., 2020). During postharvest storage, many crops produce reactive oxygen species (ROS), such as hydrogen peroxide (H_2_O_2_), hydroxyl radicals (⋅OH), superoxide anion radicals (O_2_⋅^–^), and singlet oxygen, which contribute to deteriorative changes, such as lipid peroxidation, DNA mutation, enzyme inactivation, and protein denaturation (Tian et al., 2013). Consequently, ROS generation is considered the main reason for the progression of senescence (Mittler, 2002). To resist ROS-mediated damage, plants have evolved a system that maintains a balance between ROS production and elimination involving enzymatic [superoxide dismutase (SOD; EC 1.15.1.1), catalase (CAT; EC 1.11.1.6), ascorbate peroxidase (APX; EC 1.11.1.11), and peroxidase (POD; EC 1.11.1.7)] and non-enzymatic antioxidants (Miśkiewicz et al., 2000). Despite this, ROS accumulation may exceed the antioxidant capacity, leading to membrane lipid peroxidation and impaired cellular functions (Lurie et al., 1991). Several studies have demonstrated that some plants can delay senescence by eliminating excessive ROS through enhanced antioxidant systems (Zimmermann et al., 2006; Qin et al., 2009). For instance, ultrasonic treatment was shown to effectively decrease the activities of PPO and POD and increase total antioxidant capacity, which help to inhibit the browning of fresh-cut sweet potato, thereby prolonging its postharvest shelf life (Pan et al., 2020). This indicates that antioxidant enzyme capacity is positively correlated with delayed senescence in postharvest fruits and vegetables, which can help prolong their shelf life.

Several studies have investigated the optimization of storage conditions during postharvest sweet potato storage; however, the nature of the endogenous factors that influence the storage characteristics of different sweet potato cultivars remains unclear (Fan et al., 2015; Ji et al., 2017). de Araujo et al. (2021) reported that cold-tolerant sweet potato cultivars have stronger antioxidant enzyme activities compared with those of cold-sensitive cultivars, suggestive of the important role of the antioxidant system in eliminating excessive ROS induced by low temperature. Additionally, under optimal storage temperatures, the activities of antioxidant enzymes increase in nectarines and broccoli, thereby prolonging the postharvest storage period (Zhang Z. et al., 2009; Zhao et al., 2018), while greater antioxidant enzyme activity is also associated with better storage performance in sweet potato cultivars (Tang et al., 2019). However, relatively few studies have systematically evaluated the correlation between the antioxidant system and storage property. Moreover, the screening of storage-tolerant sweet potato cultivars based on the storage property of sweet potato tubers is time-consuming and requires specific storage conditions. In this study, 10 sweet potato cultivars were selected to assess the relationship between the storage property of root tubers and the antioxidant capacity of the leaves. The sweet potato root tubers were stored at 11–15°C for 290 days, following which the rot percentage, weight loss, and α-amylase activity of the different cultivars were assessed, as were differences in antioxidant enzyme activities and ROS-related indexes in the leaves. Furthermore, the relationship between the storage property of the root tubers and the antioxidant capacity of the leaves was investigated by principal component analysis (PCA) and correlation analysis. Combination of this study provides a new method for the rapid screening of sweet potato tuber storability that involves analyzing the biochemical and physiological parameters of sweet potato leaves.

## Materials and Methods

### Plant Materials and Sample Preparation

In this study, 10 sweet potato cultivars—Xu 32, Xu 55-2, Z 15-1, Shangshu 19, Sushu 16, Yanshu 5, Hanzi, Yushu, Zhezi 3, and Yan 25—were selected from the National Sweet Potato Improvement Center (Xuzhou, Jiangsu Province, China). Undamaged root tubers of each cultivar (three replicates of 100 ± 10 tubers) were harvested in the autumn of 2013–2015 and stored for 290 days at 11–15°C. The storage property of the sweet potato cultivars was defined according to the decay percentage of the root tubers. Sweet potato cultivars with a rot percentage of less than 25% were classified as storage-tolerant; those with a rot percentage ranging from 25 to 50% were classified as above-medium storage-tolerant; those with a rot percentage between 50 and 75% were classified as medium-storable; and those with a rot percentage higher than 75% were classified as storage-sensitive (Zhang and Fang, 2006). Each cultivar was assigned a storability score based on the rot percentage. Additionally, the weight loss percentage of the sweet potato tubers was also recorded by determining the tuber weight before and after storage. Tuberous roots without pests, disease, or mechanical damage were selected for the experiment. The stem cuttings of 10 sweet potato cultivars were obtained from the National Sweet Potato Improvement Center in May 2016 and planted in the greenhouse at the Hefei University of Technology in Hefei, China, at 24°C under a 16/8-h light/dark cycle. After 2 months of growth, the mature leaves (from the second-to-top to the fifth-to-top) of 10 seedlings from each cultivar were sampled, immediately frozen in liquid nitrogen, and ground to a powder. The powder was stored at −80°C for subsequent analysis.

### Determination of α-Amylase Activity in Sweet Potato Roots

The α-amylase activity in the tuberous roots of the sweet potato cultivars was determined at 0, 30, 60, 90, 120, 150, 180, 210, 240, 270, and 290 days after storage (DAS) as described by Zhang et al. (2009). Sweet potato root samples (2.0 ± 0.05 g) were homogenized in 4 ml of 0.1 M NaAc (including 6 M CaCl_2_, pH 5.0) and centrifuged at 20,000 × *g* for 20 min. Then, 0.3 ml of the supernatant was mixed with 0.5 ml of β-limit dextrin and 0.2 ml of 10 mM NaAc and incubated at 30°C. After incubation, 5 ml of 0.01% I_2_-KI and 0.4 ml of H_2_O were added to 0.1 ml of the reaction solution, and the absorbance was determined at 560 nm. One unit of α-amylase activity was defined as the amount of enzyme needed to degrade 1 mg of β-limit dextrin per minute and was represented as U/g fresh weight (FW).

### Determination of Antioxidant Enzymes (i.e., Peroxidase, Catalase, Ascorbate Peroxidase, and Superoxide Dismutase) in Sweet Potato Leaves

The POD, CAT, APX, and SOD activities were determined following the method described by Garcìa-Limones et al. (2002). Sweet potato leaves (2.0 ± 0.05 g) were homogenized in 3 ml of enzyme extract buffer (50 mM K_2_PO_4_ pH 7.5, 1 mM ethylenediaminetetraacetic acid (EDTA), 1 mM phenylmethanesulfonyl fluoride (PMSF), 5 mM ascorbic acid (ASA), and 5% polyvinylpyrrolidone (PVP)) at 4°C and centrifuged at 12,000 × *g* for 30 min at 4°C. After centrifugation, the obtained supernatant was considered the crude enzyme solution.

The SOD activity was determined by the photochemical reduction of nitroblue tetrazolium (NBT) in the presence of riboflavin. One unit of SOD activity was defined as the amount of enzyme that inhibited the reduction of NBT by 50%; SOD activity was expressed as U/g FW. The determination of POD activity was based on the increase in absorbance at 470 nm resulting from the oxidation of guaiacol in the presence of H_2_O_2_. CAT activity was determined as the rate of decrease in absorbance at 240 nm using H_2_O_2_ as the substrate. APX activity was determined by measuring the changes in absorbance at 290 nm. The reaction system (3 ml total volume) included 50 mM phosphate buffer at pH 7.0, 15 mM ascorbic acid, 15 mM H_2_O_2_, and the appropriate amount of crude enzyme solution. One unit of POD, CAT, or APX activity was defined as an increase or decrease of 0.01 in the absorbance value per minute and was represented as U/g FW.

### Determination of Superoxide Anion Radical Production, Hydrogen Peroxide, and Malondialdehyde Content in Sweet Potato Leaves

The H_2_O_2_ content and O_2_⋅^–^ production were determined according to the methods described by Ge et al. (2017). For the determination of O_2_⋅^–^ production, 2 g of leaf powder was homogenized in 0.1 mM phosphate buffer, pH 7.8, and centrifuged at 12,000 × *g* for 30 min at 4°C; the supernatant was used for O_2_⋅^–^ determination. Each sample was divided into an experimental group and a control group. Notably, 1 ml each of the supernatant, H_3_PO_4_ buffer, and 1 mM HONH_3_Cl was mixed in a test tube and incubated at 25°C for 1 h. Then, 17 mM *p*-aminobenzenesulfonic acid and 7 mM α-naphthylamine were added and mixed, followed by incubation for an additional 20 min. Absorbance was determined at 530 nm. The O_2_⋅^–^ production rate was calculated on an FW basis in μmol⋅g^–1^⋅s^–1^. For the determination of the H_2_O_2_ content, 2 g of sweet potato leaf powder was homogenized in 3 ml of precooled acetone and centrifuged at 12,000 × *g* for 30 min. The H_2_O_2_ content was measured by determining the absorbance at 508 nm. The content of malondialdehyde (MDA), which is considered to be an indicator of the degree of plant oxidative stress, was determined according to the method described by Chen et al. (2018), with slight modifications. Sweet potato samples (2 g) were homogenized in 10 ml of 5% trichloroacetic acid and centrifuged at 12,000 × *g* for 30 min at 4°C. The absorbance of the resulting supernatant was measured at 600, 532, and 450 nm. The MDA content was calculated using the equation: MDA content (nmol/g) = [6.45 × (*A*_532_ − *A*_600_) − 0.56 × *A*_450_] × *V*_1_ × *V*_3_/(*V*_2_ × *W*), where *V*_1_, *V*_2_, and *V*_3_ indicate the total volume of the solution obtained after the reaction (ml), the volume of the extract solution used for the reaction (ml), and the volume of the extract solution (ml), respectively; *W* indicates the mass of the sample (g).

### Determination of Lipoxygenase Activity in Sweet Potato Leaves

Lipoxygenase (LOX) activity was determined by the procedure described by Surrey (1964). Sweet potato leaf powder (2 g) was homogenized in 5 ml of 0.1 M H_3_PO_4_ buffer, pH 6.8 [4% PVPP (polyvinylpolypyrrolidone) and 1% Triton X-100] and centrifuged at 12,000 × *g* for 30 min at 4°C. The obtained supernatant was considered the crude enzyme solution. The reaction solution contained 0.1 M NaAc buffer, pH 5.5, 0.01 M sodium linoleate, and the appropriate amount of crude enzyme solution. Absorbance was measured at 234 nm. One unit of LOX was defined as a decrease of 0.01 optical density (OD) value in absorbance per minute, and the results were expressed as U/g FW.

### Data Analysis

The physiological parameters of the sweet potatoes were analyzed using IBM SPSS 22.0 (IBM Corp., Armonk, NY, United States). The correlation among antioxidant enzyme activities, LOX activity, ROS-related indexes, and storage property of the different sweet potato cultivars, the heat map of the physiological parameters, and the PCA were assessed using the tools on the OmicShare platform^1^ (Gene Denovo, Guangzhou, China).

## Results

### Determination of the Storage Property of the 10 Sweet Potato Tubers

Decay percentage is one of the basic indexes used to evaluate the storage properties of sweet potatoes. In this study, 10 sweet potato cultivars (i.e., Xu 32, Xu 55-2, Z 15-1, Shangshu 19, Sushu 16, Yanshu 5, Hanzi, Yushu, Zhezi 3, and Yan 25) were selected to evaluate the decay percentage during storage. The storage properties of the different sweet potato cultivars are shown in Table 1. Xu 32 was found to be a storage-tolerant cultivar; Xu 55-2, Z 15-1, Shangshu 19, Yushu, and Zhezi 3 above-medium storage-tolerant cultivars; Sushu 16, Yanshu 5, and Hanzi medium-storable cultivars; and Yan 25 a storage-sensitive cultivar. Each sweet potato cultivar was assigned a storability score ranging from 1 (storage-sensitive) to 4 (storage-tolerant). The weight loss percentage of the tubers after 290 days of storage was also determined. The lowest weight loss percentage (18.1%) was observed in Xu 55-2 and highest (67.1%) in Sushu 16; however, no correlation was found between weight loss and storage property (Table 1).

**TABLE 1:** Storage property evaluation of 10 sweet potato cultivars, including Xu 32, Xu 55-2, Z 15-1, Shangshu 19, Sushu 16, Yanshu 5, Hanzi, Yushu, Zhezi 3, and Yan 25.

Variety	Xu 32	Xu 55-2	Z 15-1	Shangshu 19	Yushu	Zhezi 3	Sushu 16	Yanshu 5	Hanzi	Yan 25
Decay percentage	<25%	25–50%	25–50%	25–50%	25–50%	25–50%	50–75%	50–75%	50–75%	>75%
Weight loss	33.5 ± 3.2% E	18.1 ± 2.0% F	19.2 ± 3.4% F	42.5 ± 2.5% C	34.6 ± 2.7% E	37.9 ± 6.0% D	67.1 ± 4.1% A	37.6 ± 2.1% D	33.7 ± 6.2% E	51.7 ± 2.4% B
Storage level	Storage-tolerant	Above-moderate	Above-moderate	Above-moderate	Above-moderate	Above-moderate	Medium	Medium	Medium	Storage-sensitive
Storability score	4	3	3	3	3	3	2	2	2	1

### Changes in α-Amylase Activity in the Roots of the 10 Sweet Potato Cultivars During Storage

**FIGURE 1:**
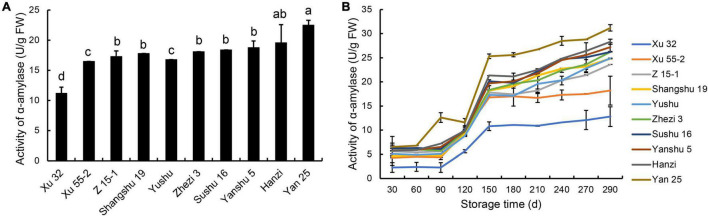
α-amylase activity in the tuberous roots of 10 sweet potato cultivars (i.e., Xu 32, Xu 55-2, Z 15-1, Shangshu 19, Sushu 16, Yanshu 5, Hanzi, Yushu, Zhezi 3, and Yan 25). **(A)** α-amylase activity in the tuberous roots of 10 sweet potato cultivars before storage and **(B)** at 30-day intervals during 290 days of postharvest storage. FW, fresh weight; d, days. Different letters above the columns in this figure and following figures stand for significant difference between two values (*p* < 0.05).

Amylase activity is responsible for starch degradation during the postharvest storage of sweet potatoes. To evaluate the correlation between α-amylase activity and storage property of the different cultivars, α-amylase activity in sweet potato tubers was determined at 30-day intervals during the 290-day storage period. As shown in Figure 1A, before storage, the lowest α-amylase activity was observed in the storage-tolerant cultivar Xu 32, while the highest was found in the storage-sensitive cultivar Yan 25. With increasing storage time, the α-amylase activity of the 10 sweet potato root tubers showed an increasing trend, peaking at 290 DAS (Figure 1B). During storage, α-amylase activity was lowest in the storage-tolerant cultivar Xu 32 and highest in the storage-sensitive cultivar Yan 25. Between days 30 and 90 of storage, the α-amylase activity of the storage-tolerant and above-medium storage-tolerant cultivars was stable and remained at a low level, whereas that of the storage-sensitive cultivar Yan 25 showed a significant increase during this storage period. These results demonstrated that α-amylase activity was lower in the storage-tolerant cultivar than in the storage-sensitive cultivar at all storage periods evaluated, and further suggested that α-amylase activity is an important indicator of the storage property of the different sweet potato cultivars.

### Analysis of the Activities of Antioxidant Enzymes in the Leaves of the 10 Sweet Potato Cultivars

**FIGURE 2:**
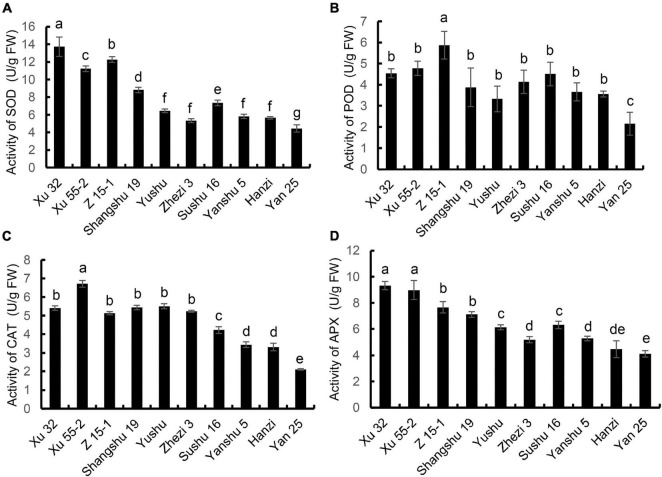
The activities of **(A)** superoxide dismutase (SOD), **(B)** peroxidase (POD), **(C)** catalase (CAT), and **(D)** ascorbate peroxidase (APX) in the leaves of 10 sweet potato cultivars (i.e., Xu 32, Xu 55-2, Z 15-1, Shangshu 19, Sushu 16, Yanshu 5, Hanzi, Yushu, Zhezi 3, and Yan 25). Data are presented as means ± SD (*n* = 3). FW, fresh weight.

The activities of antioxidant enzymes are required for ROS scavenging during sweet potato storage. Accordingly, we sought to determine whether a correlation existed between antioxidant enzyme activity in the leaves and tuber storage property. The results showed that SOD activity was highest in the storage-tolerant cultivar Xu 32 and lowest in Yan 25 (Figure 2A). SOD activity was 3.1-fold higher in Xu 32 than in the storage-sensitive cultivar Yan 25. As shown in Figure 2B, POD enzyme activity was generally consistent with the trend for SOD activity across the 10 sweet potato cultivars, with Yan 25 showing the lowest activity and Z 15-1 the highest (2.73-fold higher compared with that of Yan 25). The activities of CAT and APX in the 10 cultivars are shown in Figures 2C,D, respectively. CAT and APX activities were higher in the cultivars that showed better storage property (Xu 32, Z 15-1, Xu 55-2, Shangshu 19, Yushu, and Zhezi 3 vs. Sushu 16, Yanshu 5, Hanzi, and Yan 25). The Xu 55-2 cultivar displayed the highest CAT activity and Yan 25 the lowest. Meanwhile, APX activity was highest in Xu 32 and lowest in Yan 25. The above results indicated that the better the storage property, the higher the activities of antioxidant-related enzymes.

### Changes in Superoxide Anion Radical, Hydrogen Peroxide, and Malondialdehyde Contents and Lipoxygenase Enzyme Activity in the Leaves of the Different Sweet Potato Cultivars

**FIGURE 3:**
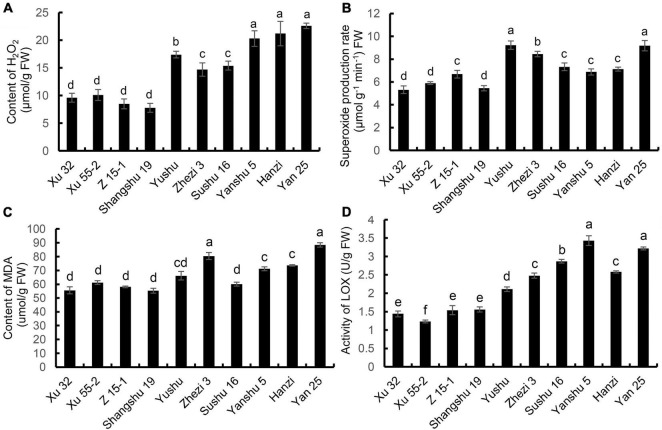
**(A)** H_2_O_2_ content, **(B)** O_2_⋅^–^ production rate, **(C)** malondialdehyde (MDA) content, and **(D)** lipoxygenase (LOX) activity in the leaves of the 10 sweet potato cultivars. Data are presented as means ± SD (*n* = 3). FW, fresh weight.

The changes in H_2_O_2_ content in the leaves of the sweet potatoes are shown in Figure 3A. The H_2_O_2_ content was lowest in the Xu 32, Xu 55-2, Z 15-1, and Shangshu 19 cultivars and highest in Yan 25. The H_2_O_2_ content in Yan 25 was 2.89-fold higher than that of Shangshu19. The H_2_O_2_ content in Xu 32, Xu 55-2, and Z 15-1, the cultivars with stronger storage performance, was significantly lower than that of Sushu 16, Yanshu 5, Hanzi, Yushu, and Zhezi 3, cultivars with reduced storage property. O_2_⋅^–^ production and MDA content showed a pattern similar to that for the H_2_O_2_ content (Figures 3B,C). The rate of O_2_⋅^–^ production was lowest in the storage-tolerant cultivar Xu 32 and highest in the storage-sensitive cultivar Yan 25 (1.72-fold that of Xu 32). The MDA content showed a gradual increase with decreasing the storage property of sweet potato. Yan 25 exhibited the highest MDA content, which was 1.6-fold that of Shangshu 19, the cultivar that displayed the lowest MDA content. As shown in Figure 3D, LOX activity was generally low in the leaves of the Xu 32, Xu 55-2, Z 15-1, and Shangshu19 cultivars and was lowest in Xu 55-2. Overall, LOX activity was higher in the leaves of the Yan 25, Sushu 16, and Yanshu 5 cultivars than in those of Zhezi 3, Sushu 16, and Hanzi. LOX activity in Yanshu 5 and Yan 25 was 2.79- and 2.62-fold, respectively, that of Xu 55-2. These findings indicated that the cultivars with better storability had lower O_2_⋅^–^, H_2_O_2_, and MDA contents, as well as lower LOX enzyme activity.

### Principal Component Analysis of Antioxidant Enzyme Activities and Reactive Oxygen Species Metabolites in the Leaves of the Different Sweet Potato Cultivars

**FIGURE 4:**
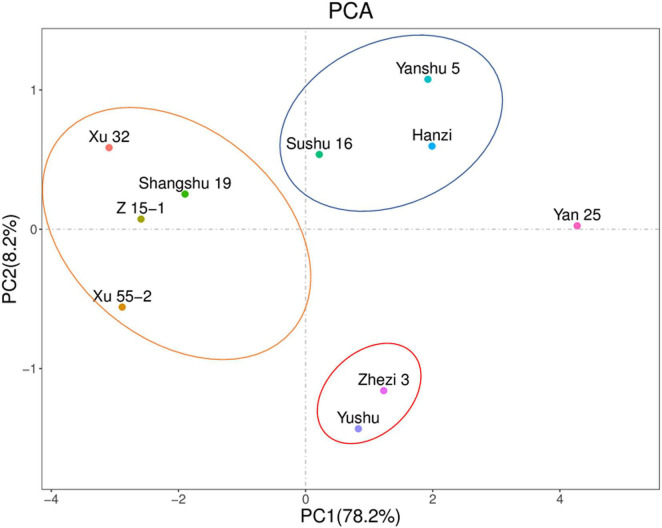
Principal component analysis based on antioxidant-related enzyme activities and reactive oxygen species (ROS) contents in 10 sweet potato cultivars (i.e., Xu 32, Xu 55-2, Z 15-1, Shangshu 19, Sushu 16, Yanshu 5, Hanzi, Yushu, Zhezi 3, and Yan 25).

The PCA showed that PC1 and PC2 accounted for 78.2 and 8.2%, respectively, of the variability in the data (Figure 4). Storage-tolerant and storage-sensitive cultivars were clearly clustered in PC1. Yanshu 5, Hanzi, and Sushu 16 were clustered together, as were Xu 32, Xu 55-2, Z 15-1, and Shangshu 19, Yushu, and Zhezi 3. The variety showing the highest positive loading on PC1 was Yan 25, and the variety that showed the lowest loading on PC2 was Yanshu 5. These observations suggested that a positive correlation exists between antioxidant capacity and storage property among the different sweet potato cultivars.

### Analysis of the Correlation Between Physiological Indexes and Storage Property in the Different Sweet Potato Cultivars

**FIGURE 5:**
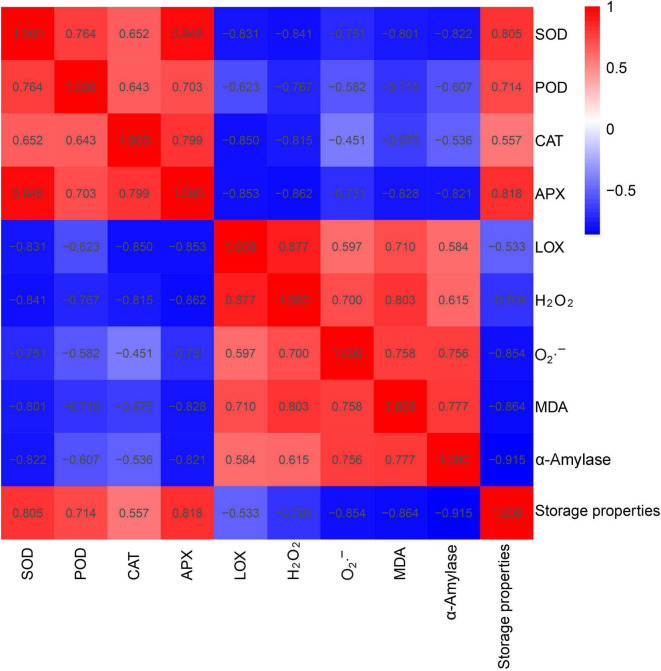
Correlation analysis among SOD, POD, APX, CAT, and LOX activities, superoxide anion (O_2_⋅^–^) production, and MDA and H_2_O_2_ contents in the leaves and storage property and α-amylase in the roots of 10 sweet potato cultivars (i.e., Xu 32, Xu 55-2, Z15-1, Shangshu 19, Sushu 16, Yanshu 5, Hanzi, Yushu, Zhezi 3, and Yan 25).

Then, the correlation among storage properties (i.e., α-amylase activity and rot percentage) of sweet potato roots and leaf parameters [i.e., ROS production (H_2_O_2_, O_2_⋅^–^, and MDA) and antioxidant enzyme activities (i.e., POD, APX, SOD, CAT, and LOX)] was analyzed (Figure 5). A positive correlation was found among storage property and POD, APX, SOD, and CAT activities, as well as among α-amylase activity, LOX activity, and O_2_⋅^–^, H_2_O_2_, and MDA contents. In addition, SOD, POD, CAT, and APX activities and storage properties were negatively correlated with LOX, α-amylase activities and O_2_⋅^–^, H_2_O_2_, and MDA contents. SOD activity was significantly and positively correlated with APX activity (*r* = 0.948) and highly and negatively correlated with H_2_O_2_ contents (*r* = −0.841). POD activity was highly and positively correlated with SOD activity (*r* = 0.764) and highly and negatively correlated with H_2_O_2_ levels (*r* = −0.767). CAT activity was highly and positively correlated with APX activity (*r* = 0.799) and negatively correlated with LOX activity (*r* = −0.850). LOX activity showed a negative correlation with APX activity (*r* = −0.853) and a positive correlation with the H_2_O_2_ content (*r* = 0.877). A negative correlation was found between H_2_O_2_ content and SOD activity (*r* = −0.841). Besides, there was a significant and negative correlation between α-amylase activity and storage property (*r* = −0.915) and a positive correlation between α-amylase activity and MDA content (*r* = 0.777). Overall, the correlation analysis indicated that the storage property of sweet potato roots is positively associated with antioxidative enzyme activity and negatively correlated with ROS metabolites in sweet potato leaves. The positive correlation detected among the activities of the antioxidant enzymes suggested that they are activated and cooperated in scavenging ROS.

### Heat Map Analysis of Antioxidant-Related Indexes and Cluster Analysis of the Relationship Among the Different Sweet Potato Cultivars

**FIGURE 6:**
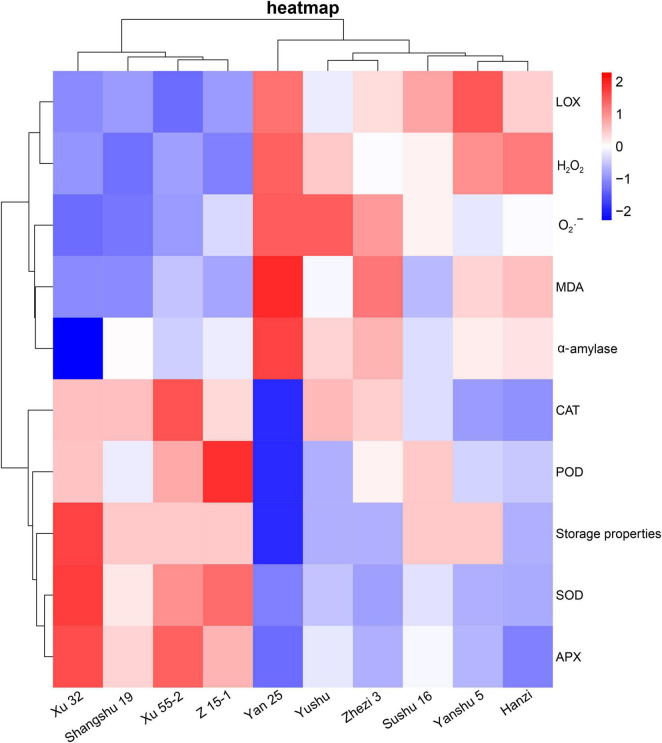
Heat map analysis of antioxidant enzyme (i.e., SOD, POD, APX, CAT), α-amylase, and LOX activities and ROS-related indexes (i.e., O_2_⋅^–^, H_2_O_2_, and MDA) in 10 sweet potato cultivars (i.e., Xu 32, Xu 55-2, Z15-1, Shangshu 19, Sushu 16, Yanshu 5, Hanzi, Yushu, Zhezi 3, and Yan 25).

To further verify the relationship between the antioxidant capacity and storage property of the different sweet potato cultivars, we generated a heat map of the antioxidant enzyme- and ROS-related indexes in the sweet potato leaves and the storage property of sweet potato (α-amylase activity). As shown in Figure 6, the sweet potato cultivars (i.e., Xu 32, Xu 55-2, Shangshu 19, and Z 15-1) with better storability were clustered together and showed higher activities of antioxidant related enzymes (i.e., POD, SOD, APX, and CAT), but significantly lower LOX and α-amylase activities, O_2_⋅^–^ production rates, and H_2_O_2_ and MDA contents relative to the storage-sensitive cultivars (i.e., Sushu 16, Yanshu 5, Hanzi, Yan 25, Yushu, and Zhezi 3). Xu 32, the cultivar with the best storage property, had the lowest α-amylase activity, while the storage-sensitive cultivar, Yan 25, had the highest.

## Discussion

Owing to its tropical origins, the tuberous roots of the sweet potato are susceptible to chilling stress (Li et al., 2018). Moreover, sweet potato decays easily during storage due to its high water content (Sugri et al., 2017). Even at appropriate storage temperatures, crops still undergo deteriorative changes resulting from the activity of internal factors, such as ROS, a key contributor to postharvest senescence (Wang et al., 2019; Guo et al., 2021). ROS can be produced in plants during many metabolic reactions, but particularly in chloroplasts and mitochondria during senescence. Throughout this process, the antioxidant defense system, comprising both enzymatic and non-enzymatic antioxidants, is activated to scavenge excessive ROS, thereby preventing cellular damage (Nie et al., 2020). Antioxidant capacity was reported to be related to the storage properties of different sweet potato cultivars (de Araujo et al., 2021), while increased antioxidant enzyme activity was found to be positively correlated with sweet potato storability (Tang et al., 2019). However, whether a correlation exists between antioxidant enzyme activity in sweet potato leaves and the storage properties of the tubers has not been determined. To address this, in this study, we evaluated whether the antioxidant capacity of sweet potato leaves is positively correlated with the storage property of sweet potato using 10 sweet potato cultivars as the experimental material.

The storability of the 10 cultivars was first determined based on decay percentage at 290 DAS. We found that Xu 32 is a storage-tolerant cultivar; Yan 25 is a storage-sensitive cultivar; Xu 55-2, Z 15-1, Shangshu 19, Yushu, and Zhezi 3 are above-medium storage-tolerant cultivars; and Sushu 16, Yanshu 5, and Hanzi are medium-storable cultivars. We also determined the weight loss percentage of the tubers at 290 DAS but found no association between weight loss and decay percentage. Accordingly, only the latter was used to categorize the storage property of sweet potato tubers. Starch constitutes an important carbohydrate reserve in tuberous roots of sweet potatoes, and amylase activity is required for starch degradation during storage (Lu et al., 2020). In this study, we found that α-amylase activity was lowest in the storage-tolerant cultivar Xu 32 and highest in the storage-sensitive cultivar Yan 25. A significant increase in α-amylase activity was observed in tubers during storage, especially in the more storage-sensitive cultivars, suggesting that a correlation exists between α-amylase activity and storage property of tubers (Figure 1), which was consistent with the results of Lu et al. (2020). We further found that sweet potato tuber storability is highly correlated with the antioxidant capacity of the sweet potato leaves, which provides a convenient means for the screening of storage-tolerant sweet potato cultivars.

Numerous studies have shown that ROS accumulates in fruit and vegetable during storage. For instance, ROS accumulation in longan postharvest leads to a gradual increase in cell membrane permeability and the destruction of cell membrane structure (Lin et al., 2005). Additionally, hydrogen sulfide treatment can increase the antioxidant capacity of strawberry, thereby prolonging its shelf life (Hu et al., 2012). Combined, these observations suggest that antioxidant capacity is intrinsic to a specific cultivar and is a key determinant of postharvest senescence. However, the relationship between the storage property of different sweet potato cultivars and the antioxidant capacity of sweet potato leaves still needs further investigation. In this study, we found that antioxidant enzyme (i.e., CAT, POD, APX, and SOD) activity in the leaves of the storage-tolerant cultivars Xu 32, Xu 55-2, and Z 15-1 remained at higher levels compared with those of the storage-sensitive cultivars Yan 25, Sushu 16, Yanshu 5, and Hanzi, whereas the opposite was seen for LOX activity. Besides, the storage-tolerant cultivar Xu 32 and the above-moderate storage-tolerant cultivars Xu 55-2 and Shangshu 19 contained lower levels of ROS metabolites when compared with those of the storage-sensitive cultivar Yan 25, all of which suggested that antioxidant capacity is positively correlated with sweet potato storability.

**FIGURE 7:**
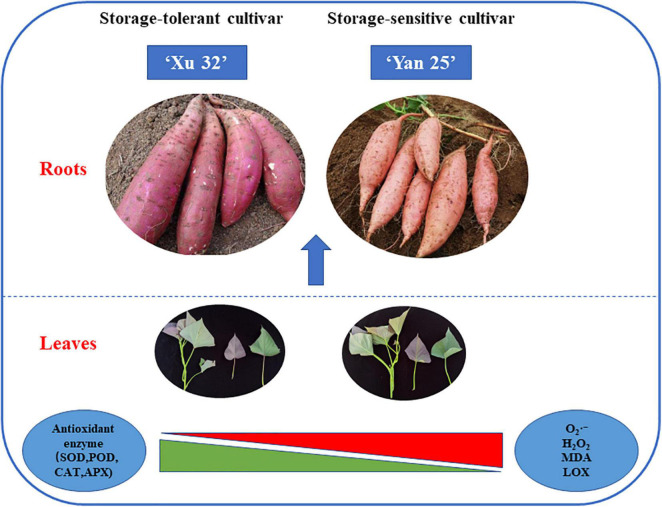
Schematic diagram showing that ROS and MDA levels; LOX, SOD, POD, APX, and CAT activities in sweet potato leaves are highly associated with the storage property of different sweet potato tubers.

Furthermore, correlation and heat map analysis showed that there was a prominent association between the antioxidant capacity of sweet potato leaves and the storage property of sweet potato tubers, while antioxidant enzyme activity was negatively correlated with the levels of ROS metabolites and positively correlated with storage property. Moreover, α-amylase activity was found to be negatively correlated with storage property, suggesting that α-amylase activity is also a valuable index for evaluating the storage potential of sweet potato tubers. PCA indicated that sweet potato cultivars with similar antioxidant enzyme activities, such as Xu 32, Xu 55-2, Z 15-1, and Shangshu 19, were clustered together, as were Yanshu 5, Hanzi, and Sushu 16 (Figure 3). Overall, these results were consistent with the storage properties of the different cultivars and sweet potato varieties with similar antioxidant enzyme activities. The growth environment, soil, fertilizer and water management, temperature, light, and other external conditions can all affect the storage performance of sweet potato, while the ecological environment can significantly affect sweet potato quality (Yan et al., 2017). Rosenthal and Jansky (2008) reported that antioxidant activity in potato growing in a high-yield production environment was usually the highest and increased during storage, indicative of the importance of antioxidant enzymes for potato storability. Additionally, ultrasound treatment can inhibit the browning of fresh-cut sweet potatoes by reducing PPO and POD activities while improving total antioxidant capacity (Pan et al., 2020). Moreover, low-temperature conditioning at 10°C can induce antioxidant enzyme activity in tuberous roots and protect tubers from chilling injury when subjected to subsequent cold storage at 4°C (Li et al., 2018). Together, these findings suggest that the antioxidative enzyme system is critical for protecting the sweet potato from postharvest senescence and decay. As shown in Figure 7, antioxidant enzymes are required for maintaining ROS metabolic balance, while accumulated ROS may negatively influence the storage property of sweet potato tubers.

Overall, this study provides strong evidence that the antioxidant capacity of leaves in different sweet potato cultivars is positively correlated with their storability. We further found that α-amylase activity in sweet potato tubers is negatively correlated with storage property, suggesting that α-amylase activity may represent a valuable index for evaluating the storage potential of sweet potato tubers. Finally, given that the characterization of storage property in different sweet potato cultivars is a time-consuming process, this study provides a convenient means for evaluating the storage properties of sweet potatoes by measuring the antioxidant capacity in sweet potato leaves.

## Data Availability Statement

The original contributions presented in the study are included in the article/supplementary material, further inquiries can be directed to the corresponding author/s.

## Author Contributions

H-HS, K-DH, G-FY, and HZ conceived and designed the experiments. H-HS, Z-LZ, and D-LZ performed the experiments. Z-LZ, D-LZ, HZ, and X-YC analyzed the data. K-DH, G-FY, and H-HS wrote the manuscript. K-DH, G-FY, and ZH interpreted the data and revised the manuscript. All authors contributed to the article and approved the submitted version.

## Conflict of Interest

The authors declare that the research was conducted in the absence of any commercial or financial relationships that could be construed as a potential conflict of interest.

## Publisher’s Note

All claims expressed in this article are solely those of the authors and do not necessarily represent those of their affiliated organizations, or those of the publisher, the editors and the reviewers. Any product that may be evaluated in this article, or claim that may be made by its manufacturer, is not guaranteed or endorsed by the publisher.

## Footnotes

1
https://www.omicshare.com/
